# Schnitzler's Syndrome: A Case Report

**DOI:** 10.1155/2013/956464

**Published:** 2013-06-09

**Authors:** Gabriel Tinoco, Rehan Kanji, Deepthi Moola

**Affiliations:** ^1^Division of Hospital Medicine, Department of Medicine, University of Miami Miller School of Medicine, 1120 NW 14th Street, Suite 1185 CRB, Miami, FL 33136, USA; ^2^American University of Antigua, 1 Battery Park Plaza, New York, NY 10004, USA; ^3^Department of Obstetrics and Gynecology, TriHealth, 619 Oak Street, Cincinnati, OH 45206, USA

## Abstract

Schnitzler's syndrome is an extremely rare entity that poses a challenge for the clinician not only due to its difficult diagnosis but also due to its management. In this article we report a new case and briefly review the current treatment options.

## 1. Introduction

Schnitzler's syndrome is an extremely rare and not completely understood entity. It is characterized by a chronic recurrent urticarial rash with leukocytoclastic vasculitis, monoclonal IgM gammopathy, intermittent fevers, arthralgia, bone pain, lymphadenopathy, skeletal hyperostosis, and occasionally hepato- or splenomegaly [1–7].

To date only around 100 cases [1, 2, 8] have been reported since the first described case in 1972 [9].

This autoinflammatory syndrome clinically presents with a diverse constellation of symptoms making its initial diagnosis very challenging. Its early recognition is key due to the increased risk of lymphoproliferative disorders (10–15% per year [2]) and its tremendous impact on the quality of life of the patients with this entity.

Due to its obscure pathophysiology, the treatment remains not well defined, being steroids the first line of treatment [3, 10]. However different treatment modalities, with different range of responses, have been reported including NSAIDs, [3] thalidomide [11, 12], methotrexate [13], cyclosporine [14], cyclophosphamide [15], psoralen plus ultraviolet [16], interferon [17], and recently biological therapies such as TNF*α* blockade [18, 19], Anakinra [12, 20–24], Rituximab [25, 26], and Tocilizumab [27, 28] (Table 2).

## 2. Case Report 

A 49-year-old Caucasian female presented with generalized arthralgias and recurrent diffuse pruritic maculopapular lesions over her face, torso, and upper extremities. The patient suffered outbreaks with variable intensity almost in a daily basis over the past five years with no identifiable triggering factors.

The patient mentioned that the lesions cleared up in 24 hours, leaving no marks or scars, while new lesions appeared daily.

Her past medical history was significant for transfusion related hepatitis C, diabetes mellitus type 2, depression, chronic pancreatitis, and malnutrition. 

Previous surgeries included pancreatic cyst removal at age 11, tonsillectomy/adenoidectomy, tubal ligation, and appendectomy.

She had a 35 pack/year history of smoking, with remote history of inhaled crack cocaine use. She denied alcohol or other illicit drug use.

Review of systems revealed unintended weight loss of about 20 pounds over a 6-month period, diffuse abdominal pain, generalized weakness, night sweats, and subjective recurrent low-grade fevers not associated with the onset of her skin lesions.

Her family history was noncontributory (father deceased at the age of 70 because of heart disease, mother had diabetes mellitus and coronary artery disease, and her brother and daughter had good health).

**Figure 1:**
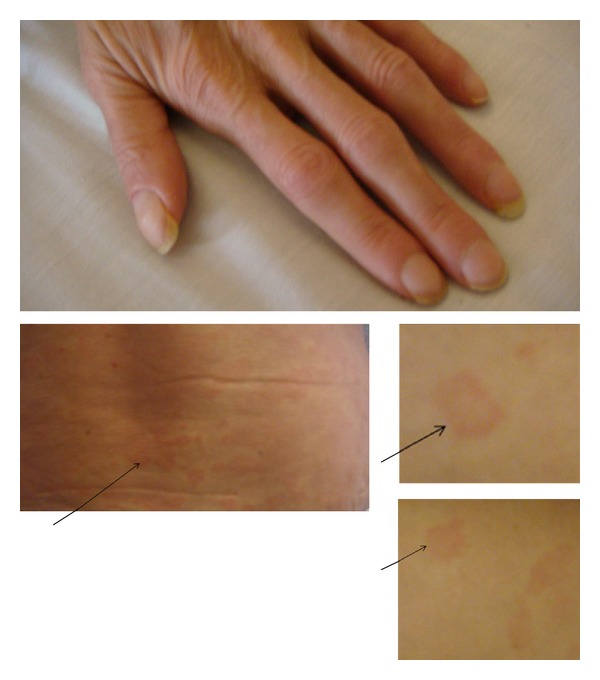
Urticaria as seen on the skin of the back, thigh, and arm of patient (from left to right).

The initial physical examination revealed diffuse pruritic maculopapular lesions over her face, thorax, and upper extremities (Figure 1), sparing her palms, soles, and mucous membranes. Also mild hepatosplenomegaly was found.

Her laboratory studies showed marked leukocytosis of 48.6 with neutrophils of 97.8% with elevated D-dimer and erythrocyte sedimentation rate (ESR) (Table 1).

She was started on broad-spectrum antibiotics that were discontinued 72 hours after the admission when infections were ruled out. 

Antinuclear antibodies, rheumatoid factor, anticitrullinated peptide antibodies, human immunodeficiency virus enzyme-linked immunosorbent assay (ELISA), Venereal Disease Research Laboratory (VDRL), hepatitis B surface antigen (HBsAg), hepatitis B core antigen (HBcAg), hepatitis B e antigen (HBeAg), and cryoglobulins were found to be negative or within normal limits.

Serum protein electrophoresis showed monoclonal gammopathy; SPEP showed gamma region asymmetric spike consistent with monoclonal gammopathy. UPEP showed oligoclonal gammopathy involving IgG, IgM kappa, and lambda (IgM: 552 mg/L).

Biopsy of her skin lesions showed neutrophil rich cells admixed with eosinophils consistent with chronic urticaria. A right groin excisional lymph node biopsy showed reactive lymphoid hyperplasia with plasmacytosis.

Bone marrow biopsy showed a hypercellular marrow with plasmacytosis and myeloid predominance and was reported as negative for malignancy. 

Based on the clinical presentation and laboratory results the diagnosis of Schnitzler syndrome was made. As proposed by Lipsker et al. [3] the diagnosis of this entity requires 2 major criteria and a minimum of 2 minor criteria. She had 2 major criteria (chronic urticarial rash/intermittent fever and monoclonal IgM) plus at least 5 minor criteria (arthralgias, bone pain, lymphoadenopathy, hepato- and/or splenomegaly, and elevated ESR and/or leukocytosis).

**Figure 2:**
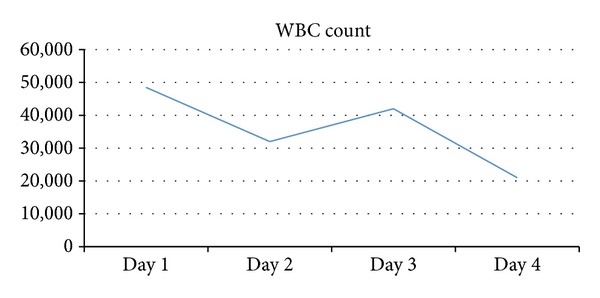
Progression of WBC count throughout the hospital course.

She was started on high dose of corticosteroids, nonsteroidal anti-inflammatory drugs, and antihistamines with partial remission of her symptoms and she was discharged home (Figure 2).

Anakinra was added to her regimen for several weeks with an excellent response; however due to economical constrains treatment was discontinued, and her symptoms partially recurred.

## 3. Conclusions 

Schnitzler's syndrome is an underdiagnosed disease with only around 100 cases reported in the literature. Having eastern European origin [1, 2, 7, 8] and being a woman (women are more frequently affected than man (1.6 : 1) [2]) are strong associations to this entity.

The mortality risk is not increased in patients with Schnitzler's syndrome (survival of 94% after 15 years of the diagnosis) [2]; however there is a well-established increased risk of malignancy (specially lymphoproliferative disease such as Waldenström macroglobulinemia and IgM myeloma) of 10-year risk of 15% [2, 31]. 

Systemic steroids remain as the first line of treatment [3, 8, 10], being effective to less than a half of the patients [8]. The data available demonstrated that the treatment of Schnitzler syndrome is not standardized, and there are promising results with new biological therapies such as the interleukin-1 receptor antagonist Anakinra [12, 20–24], Rituximab [25, 26], and Tocilizumab [27, 28]. 

## Figures and Tables

**Table:** (a)

WBC	48.6
Differential	97.8% *N*
Hemoglobin	13
Hematocrit	40.9
Platelets	321

**Table:** (b)

D-dimer	565	BNP	489
CK	<20	CKMB	<0.3
Trop I	<0.1	Lactic Acid	2.6
Amylase	<30	Lipase	42
Alk. phos	259	AST/ALT	11/14
PT	15.8	PTT	37.3
INR	1.3	ESR	80

**Table:** (c)

Sodium	125	Potassium	4.9
Chloride	92	Bicarbonate	27
BUN	16	Creatinine	0.6
Glucose	230	Calcium	8.1
Phosphorous	4.0	Magnesium	1.5

**Table 2:** Common therapeutic strategies for Schnitzler's syndrome.

Treatment	Efficacy	References
Corticosteroids	High doses may achieve complete remission in about 39% of the cases.	[1–3, 7, 8, 11, 12, 14–17, 29, 30]
NSAID's	Partial or temporary remission of the symptoms in about 15% of the cases.	[2, 3, 6–8, 11, 12, 29, 31]
Antihistamines	Partial or temporary remission of the symptoms.	[2, 3, 8, 12, 16, 29, 30]
Cyclophosphamide, methotrexate	Partial remission in occasional cases.	[2, 8, 13, 15]
Anakinra	Complete remission in few cases.	[2, 8, 12, 20–24]
